# Comparison of GLP-1 Analogues versus Sitagliptin in the Management of Type 2 Diabetes: Systematic Review and Meta-Analysis of Head-to-Head Studies

**DOI:** 10.1371/journal.pone.0103798

**Published:** 2014-08-04

**Authors:** Tiansheng Wang, Zhuoyue Gou, Fei Wang, Manling Ma, Suo-di Zhai

**Affiliations:** 1 Department of Pharmacy Administration and Clinical Pharmacy, Peking University Health Science Center, Beijing, China; 2 Department of Pharmacy, First Affiliated Hospital of Harbin Medical University, Harbin, China; 3 Department of Pharmacy Practice, School of Pharmacy, University of Connecticut, Storrs, Connecticut, United States of America; 4 Department of Pharmacy, Peking University Third Hospital, Beijing, China; University of Lancaster, United Kingdom

## Abstract

**Background:**

Incretin–based therapies which include glucagon-like peptide-1 (GLP-1) receptor agonists and dipeptidyl peptidase-4 (DPP-4) inhibitors are recommended by several practice guidelines as second-line agents for add-on therapy to metformin in patients with type 2 diabetes (T2DM) who do not achieve glycemic control with metformin plus lifestyle interventions alone. The purpose of this study is to perform a systematic review with meta-analysis of existing head to head studies to compare the efficacy and safety of GLP-1 analogues with DPP-4 inhibitors.

**Methods:**

We performed a systematic review and meta-analysis of head-to-head studies to compare GLP-1 analogues with DPP-4 inhibitors in the management of type 2 diabetes. A random effects model was selected to perform the meta-analyses, results were expressed as weighted mean differences for continuous outcomes and relative risks for dichotomous outcomes, both with 95% confidence intervals, and with I^2^ values and P values as markers of heterogeneity.

**Results:**

Four head-to-head randomized controlled studies with 1755 patients were included. Compared to sitagliptin, GLP-1 analogues are more effective in reducing HbA1C (weight mean difference −0.41%, 95% CI −0.51 to −0.31) and body weight (weight mean difference −1.55 kg, 95% CI −1.98 to −1.12). Conversely, GLP-1 analogues are associated with a higher incidence of gastrointestinal adverse events compared to sitagliptin: nausea (relative risk 3.14, 95% CI 2.15 to 4.59), vomiting (relative risk 2.60, 95% CI 1.48 to 4.56), diarrhea (relative risk 1.82, 95% CI 1.24 to 2.69), and constipation (relative risk 2.50, 95% CI 1.33 to 4.70).

**Conclusions:**

The result of this meta-analysis demonstrates that compared to sitagliptin, GLP-1 analogues are more effective for glycemic control and weight loss, but have similar efficacy in reducing blood pressure and lipid parameters, however, GLP-1 analogues are associated with a higher incidence of gastrointestinal adverse events and a similar incidence of hypoglycemia compared to sitagliptin.

## Introduction

In patients with T2DM, the incretin effect is reduced or in some cases, absent [1]. Targeting the incretin system has become an important therapeutic approach to lowering elevated plasma glucose levels in type 2 diabetes. Incretin hormones are intestinally derived peptides that play a role in the maintenance of glycemic control. There are two naturally occurring incretin hormones, glucagon-like peptide-1 (GLP-1) and glucose-dependent insulinotropic polypeptide (GIP), which are responsible for insulin release in a glucose-dependent manner, however, other physiological effects between these two hormones differ significantly in regards to glucagon suppression and effects on satiety and body weight. Both GLP-1 and GIP have a short half-life because of their rapid inactivation by DPP-4 enzyme. GLP-1 has multiple physiological effects that make it a more attractive candidate for treatment of T2DM. Administration of pharmacological levels of GLP-1 analogues resistant to DPP-4, not only increases insulin secretion while inhibiting glucagon release in a glucose-dependent fashion, but also delays gastric emptying and suppresses food intake [1]–[3].

Current GLP-1 analogues approved for use in the United States and the European Union include: exenatide twice daily [4], exenatide once weekly [5], liraglutide once daily [6], lixisenatide once daily (not approved in the U.S.) [7] and albiglutide once weekly [8], which are all delivered through subcutaneous injection and initial dose titration is required to improve gastrointestinal tolerance. The DPP-4 inhibitors reduce endogenous GLP-1 degradation, by inhibiting DPP-4 enzyme, thereby providing physiological levels of GLP-1 [9]. Currently available DPP-4 inhibitors include sitagliptin [10], saxagliptin [11], linagliptin [12], vildagliptin (not approved in the U.S.) [13], and alogliptin [14]. DPP-4 inhibitors are available orally and there is no need for dose titration when initiating treatment [15].

GLP-1 receptor agonists and DPP-4 inhibitors are included in the 2012 American Diabetes Association (ADA)/European Association for the Study of Diabetes (EASD) and 2013 American Association of Clinical Endocrinologists (AACE) guidelines as second-line therapy for patients who do not achieve glycemic control with metformin therapy and lifestyle modifications alone. The National Institute for Health and Clinical Excellence (NICE) clinical guideline for T2DM recommends adding a DPP-4 inhibitor instead of a sulfonylurea as second line treatment to first line metformin when there is a considerable hypoglycemia risk or a sulfonylurea is contraindicated or not tolerated [16]. As both GLP-1 analogues and DPP-4 inhibitors are increasingly used in the management of T2DM (more often in combination therapy with metformin) [17], one important question that may arise is which one of the two drug classes is more favorable as a second-line treatment of T2DM [18], [19].

A meta-analysis of placebo-controlled clinical trials assessing the safety and efficacy of incretin-based therapy showed that the GLP-1 analogues are more effective in lowering blood glucose and weight loss, whereas sitagliptin lowers blood glucose levels to a lesser degree and are weight neutral [20]: the results showed that unadjusted HbA1c changes for exenatide, liraglutide, and sitagliptin are −0.75% (−0.83, −0.67), −1.03% (−1.16, −0.90), and −0.79% (−0.93, −0.65), respectively; and unadjusted weight changes for exenatide, liraglutide, and sitagliptin are −1.10 kg (−1.32, −0.88), −0.82 kg (−1.92, 0.27), and 0.60 kg (0.33, 0.87), respectively. However, a major potential pitfall of this meta-analysis was the use of unadjusted data which introduces confounding factors that may affect the end outcomes of the study [18]. Therefore, head-to-head comparative studies are needed to compare the efficacy and safety of GLP-1 analogues and DPP-4 inhibitors directly and accurately. Pinelli et al., performed a meta-analysis to compare long acting GLP-1 analogues with short acting exenatide and sitagliptin [21]; only one study included in this meta-analysis directly compared the 2 classes of incretin-based therapies. Other reviews in the literature have reported on the efficacy and safety of GLP-1 analogues and DPP-4 inhibitors [22]–[27]; however, to our know knowledge, no meta-analysis of head to head studies comparing the 2 classes of incretin therapy has been published. Thus we performed a systematic review with meta-analysis of existing head-to-head studies comparing the efficacy and safety of GLP-1 analogues with the DPP-4 inhibitors [28]–[37] to provide a more accurate and rigorous statistical analysis.

## Methods

The main objective of this meta-analysis was to assess the efficacy and safety of GLP-1 analogues compared to the DPP-4 inhibitors in the management of patients with T2DM. Outcome measures included glycemic control, weight loss, changes in blood pressure, lipid profile, and common adverse events. We followed the methods specified in the Cochrane Handbook for Reviews on Interventions [38].

### Data sources

Eligible trials were identified through electronic and manual searches. Electronic searches were performed in Medline, Embase, Cochrane Library, and Clinicaltrials.gov from its inception until January 2014. The search was limited to English articles. In the Medline database, we used the search strategy for “exenatide”, “liraglutide”, “lixisenatide” or “glucagon-like peptide-1”; and “dipeptidyl peptidase-4” or “sitagliptin” or “saxagliptin” or “linagliptin” or “alogliptin” or “vildagliptin”; and “Randomized Controlled Trial” or “RCT” or “random”. These terms were adjusted to fit the requirements specified in the remaining databases. Manual searches included scanning of reference lists in relevant papers, conference proceedings. Literature search was performed by two independent reviewers (ZG and TW).

### Study selection

Electronic searching results were imported in a reference management software (Mendeley Desktop 1.10.1). After deleting the duplicate results, two reviewers (TW and ZG) independently screened all titles and abstracts and investigated full texts for eligible studies. Studies were included if they met the following inclusion criteria: (1) designed as randomized controlled trials; (2) head-to-head trials comparing GLP-1 analogues and DPP-4 inhibitors as monotherapy or add-on therapy to metformin; (3) Enrollment of patients with type 2 diabetes only; (4) duration of intervention of at least 12 weeks.

### Data extraction

Two authors extracted data independently (TW, ZG) and any discrepancies were resolved by consensus. From each study we extracted study characteristics (author identification, year of publication, National Clinical Trial (NCT) number, name of the trial if applicable, study location, sample size for each group, duration of intervention); participants’ baseline characteristics (age, sex, race, duration of type 2 diabetes, HbA1C, body weight, body mass index (BMI)); and pre-specified outcomes of efficacy and safety. Our primary outcome was glycemic control as measured by the change in HbA1C from baseline to end of study. Secondary efficacy outcomes included changes in body weight, fasting and postprandial plasma glucose values, percentage of patients achieving a HbA1C <7%, blood pressure (systolic and diastolic) and lipid parameters (total cholesterol, high-density lipoprotein (HDL), low-density lipoprotein (LDL), and triglyceride levels). Safety outcomes extracted included withdrawal rates from any adverse events that documented incidence of hypoglycemia, nausea, vomiting, diarrhea, constipation, urinary tract infection (UTI), upper respiratory infection (URTI), nasopharyngitis, and headache based on their clinical relevance or relatively high frequency in previous studies [24], [26], [39]. An attempt to contact the investigators was made to clarify or request additional information if appropriate.

### Quality assessment

Cochrane Collaboration’s risk of bias tool was used to assess risk of bias in randomization methods (allocation sequence generation and allocation concealment), blinding (of participants, personnel and investigators), completeness of outcome data, reporting of data and other biases [40]. We summarized the risk of bias of all six domains to produce an overall risk of bias. The following judgments were used: low risk, high risk, or unclear (either lack of information or uncertainty over the potential for bias). Two authors (TW, ZG) independently assessed the risk of bias and resolved disagreements by consensus with a third author (SZ) to resolve disagreements if necessary.

### Data analysis

Meta-analysis was conducted with the Review Manager (Revman Version 5.2, Copenhagen, Denmark). The Cochran Q χ^2^ test and I^2^ statistic were used to assess heterogeneity among studies. As the observed effect estimates can vary across studies because of real differences in the treatment effect in each study as well as sampling variability, random effects model was selected. Results of the meta-analysis were expressed as weighted mean differences for continuous outcomes and relative risks for dichotomous outcomes, both with 95% confidence intervals, and with I^2^ values and P values as markers of heterogeneity. I^2^ values of 30–60% and over 75% represent moderate and considerable heterogeneity, respectively [41]. If a standard deviation was not provided in a study, this was calculated from the sample size and the standard error or the 95% confidence interval (CI); when calculation was not feasible, standard deviation was imputed from other studies [42]. Data for intention to treat (all participants randomized) or modified intention to treat (all randomized participants who received intervention and had at least one measurement after baseline) populations were used when these were available either in a published paper or trial registries (www.clinicaltrial.gov).

We performed subgroup analyses to examine different interventions (exenatide, liraglutide). The mean difference or relative risk was further evaluated by classifying each study into one of these categories. Initial sensitivity analyses included repeating all meta-analyses using fixed effect models. The results of these analyses were only reported if the conclusions differed. A sensitivity analyses was performed to evaluate the influence of each study in each main analysis through omitting one study at a time to assess whether the pooled estimates were excessively influenced by any single study. Publication bias was examined by Egger’s test if >10 studies were included in the analysis of the primary outcomes [43]. Meta-regression was performed to investigate the characteristics of different studies if >10 studies were included [40].

## Results

### Literature searches and study inclusion

**Figure 1:**
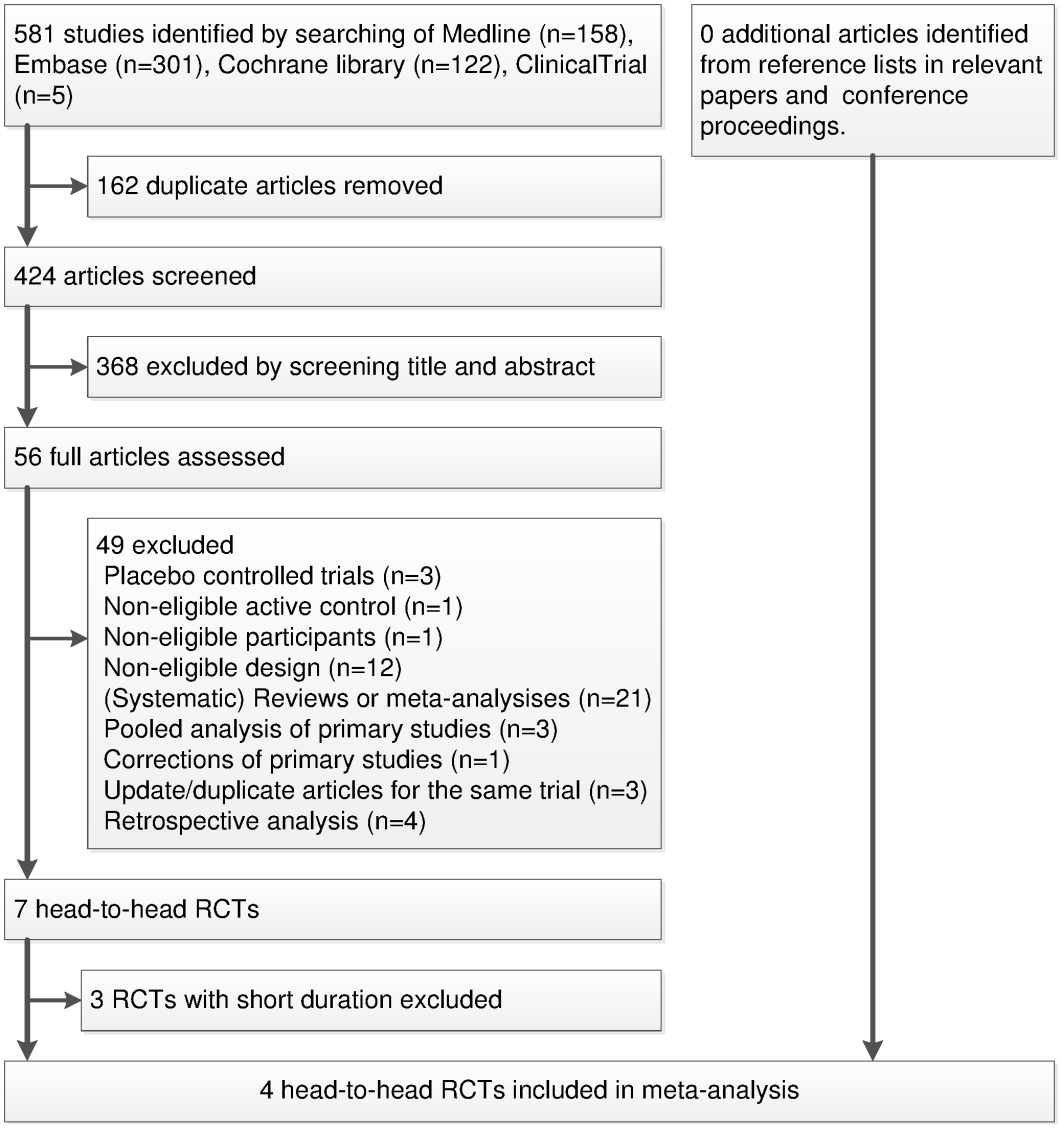
Article selection diagram for meta-analysis.

The electronic searches identified 581 potentially relevant articles. After excluding duplicates and studies that did not meet our inclusion criteria, 7 head to head RCTs were identified [28]–[30], [32]–[34], [36] and 4 RCTs were included in the meta-analysis [30], [32], [34], [36] (Figure 1). All 4 trials were published as full paper articles between 2010 and 2013 (Table 1). We did not obtain any eligible studies through manual searches. The patient characteristics at baseline were similar across trials. Mean body mass index ranged from 31.4 to 32.7 kg/m^2^ in the GLP-1 groups and 31.8 to 32.6 kg/m^2^ in the sitagliptin groups. Mean values of HbA1C at baseline ranged from 8.1% to 8.5% for GLP-1 analogues and 8.2% to 8.5% for sitagliptin groups, respectively. All 4 RCTs were multicentered (mean number of clinical sites 50) and multinational (most were done in US and Europe). Three RCTs were of short duration ranging from 12 to 26 weeks and one RCT had a 26-week main study phase [34] with a 26-week extension phase [35], thus the data for the main phase of the study was used for meta-analysis to minimize the heterogeneity. One trial intensified therapy by increasing the dose for GLP-1 group and adding glimepiride to sitagliptin groups after 12 weeks [32], so only data at 12 weeks were included for meta-analysis, and standard deviation of this trial was imputed from the other trial assessing liraglutide [34] with a reasonably high standard deviation [42].

**Table 1:** Characteristics of head to head RCT studies of GLP-1 analogues and Sitagliptin included in the meta-analysis.

Source(NCT number)	Location	Study type	StudyDuration,(primarystudy +extension)weeks	Backgroundtherapy	Treatment	No. ofpatient	Woman(%)	White(%)I/C	BaselineHbA1Clevel (%)I/C	Age (years)I/C	Duration ofT2DM I/C	Weight(kg) I/C	BMI (kg/m^2^)I/C
**Bergenstal** **2010^30^** **(NCT00637273)**	72 hospitals andclinics in theUSA,India, andMexico	double-blind,double-dummyRCT	26	Metformin	I: Exenatide2 mg QW	I: 160	I: 44	I: 33	I: 8.3 (1.0)	I: 52 (10)	I: 6 (5)	I: 89 (20)	I: 32 (5)
				Metformin	C: Sitagliptin100 mg QD	C: 166	C: 48	C: 30	C: 8.3 (1.1)	C: 52 (11)	C: 5 (4)	C: 87 (20)	C: 32 (5)
**Charbonel** **2013^32^** **(NCT01296412)**	111 clinical sitesin 21 countries	Open-label RCT	12^a^+14	Metformin	I: Liraglutide1.2 mg QD	I: 327	I: 45	I: 84	I: 8.1 (0.9)	I: 57.6 (10.8)	I: 7.6 (4.8)	I: 92.1 (20.4)	I: 32.7 (6.1)
				Metformin	C: Sitgaliptin100 mg QD	C: 326	C: 45	C: 86	C: 8.2 (1.1)	C: 56.9 (10.0)	C: 8.2 (6.2)	C: 91.0 (20.5)	C: 32.6 (5.9)
**Pratley** **2010^34^** **(NCT00700817)**	83 clinical sitesin 14 countries	parallel-group,open-label RCT	26^b^	Metformin	I_1_: Liraglutide1.2 mg QD	I_1_: 221	I_1_: 48.4	NR	I_1_: 8.4 (0.8)	I_1_: 55.9 (9.6)	I_1_: 6.0 (4.5)	NR	I_1_: 32.6 (5.2)
				Metformin	I_2_: Liraglutide1.8 mg QD	I_2_: 218	I_2_: 47.5	NR	I_2_: 8.4 (0.7)	I_2_: 55.0 (9.1)	I_2_: 6.4 (5.4)	NR	I_2_: 33.31 (5.1)
				Metformin	C: Sitagliptin100 mg QD	C: 219	C: 45.2	NR	C: 8.5 (0.7)	C: 55.0 (9.0)	C: 6.3 (5.4)	NR	C: 32.6 (5.4)
**Russell-Jones** **2012^360^** **(NCT00676338)**	62 clinical sitesin 22 countries	Double-blindRCT	26	None	I: Exenatide2 mg QW	I: 248	I: 44	I: 68.1	I: 8.5 (1.2)	I: 54 (11)	I: 2.7 (3.2)	I: 87.5 (18.9)	I: 31.4 (5.3)
				None	C: Sitgaliptin100 mg QD	C: 163	C: 42.3	C: 69.3	C: 8.5 (1.3)	C: 52 (11)	C: 2.7 (3.7)	C: 88.7 (18.7)	C: 31.8 (5.4)

I, intervention; C, control; BMI, body mass index; FPG, fasting plasma glucose; EXE, exenatide; SIT, sitagliptin; GLI, glimepiride, QW, every week, QD, every day; NR, not reported.

*the number is obtained by contacting the author;

a Charbonel et al 2013 [32], only data from 1^st^ 12 weeks were used to because after 12 weeks, patients on sitagliptin with an HbA_1c_ ≥7.0% and FFG >6.1 mmol/l had glimepiride added to their treatment regimen for an additional 14 weeks, and patients on liraglutide 1.2 mg/d with an HbA_1c_ ≥7.0% had the liraglutide dose, as per label, titrated up to 1.8 mg/day.

b Data of week 26 is obtained from Prateley 2010 [34].

All 4 trials directly compared GLP-1 analogues groups with sitagliptin. Oral sitagliptin 100 mg daily was the only dose assessed in the control groups. The GLP-1 analogues were given as once weekly exenatide (2 mg) in 2 RCTs, and once daily liraglutide 1.2 mg in one RCT (Table 1); another RCT compared liraglutide 1.2 and 1.8 mg/day with sitagliptin, respectively [34], the outcome data of liraglutide 1.2 mg was used for major meta-analysis to minimize heterogeneity; we also repeated analysis with the data of liraglutide 1.8 mg and reported the results if they differed from the major meta-analysis. The efficacy and safety data were shown in Table S1 and Table S2, respectively.

### Quality of bias control

**Figure 2:**
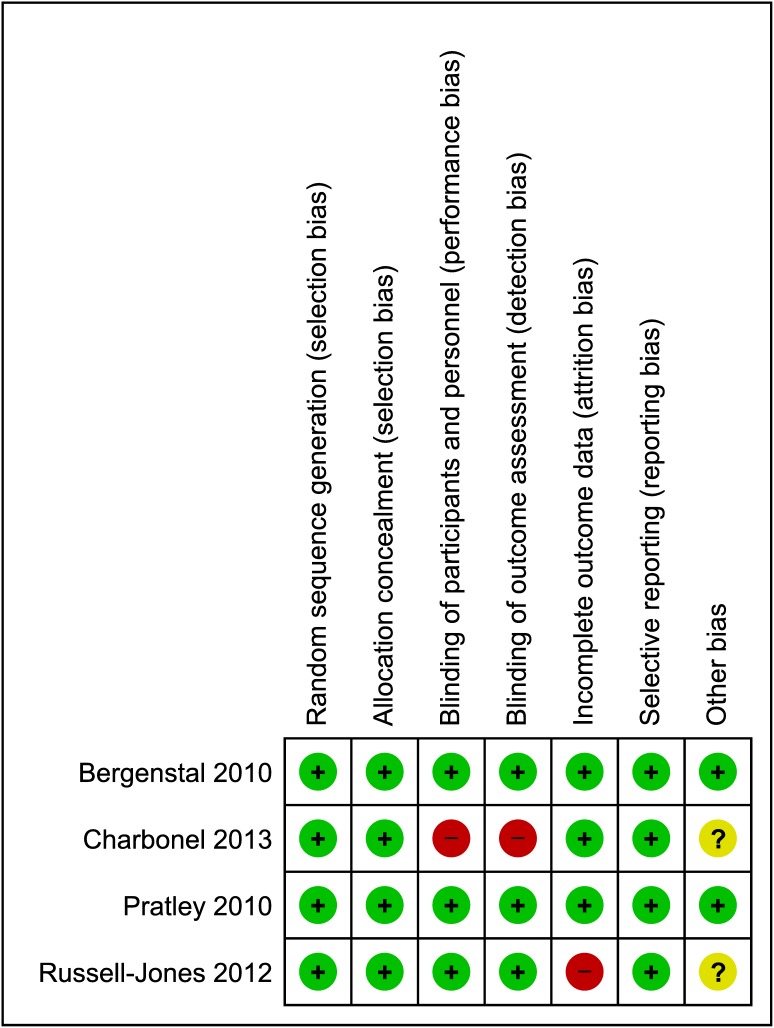
Risk of bias summary. +, Low risk of bias; − high risk of bias; ?, unknown risk of bias. Risk of bias assessment for random sequence generation and allocation concealment is performed at the study level. Risk of bias assessment for blinding of participants and personnel, incomplete outcome data, selective reporting, and overall risk of bias are for the primary outcome (change in HbA1c).

The randomization methods were described as adequate in 4 trials (Figure 2). None of the trials found differences in the baseline characteristics of participants between the GLP-1 analogue and sitagliptin groups. All 4 trials described random sequence generation and allocation concealment, reported clinically relevant outcome measures, and undertook sample size calculations [30], [32], [34], [36]. Three trials provided a clear description of losses to follow-up and accounted for patients with missing data in the analyses [30], [32], [34]. None of the included trials were terminated prematurely.

### Glycemic control

**Figure 3:**
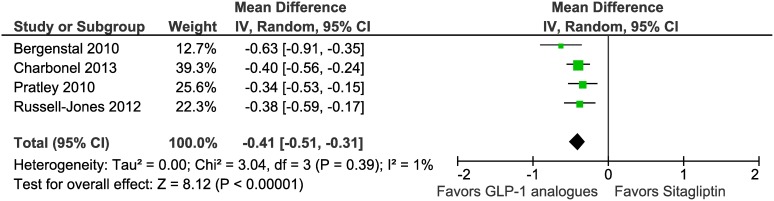
Meta-analysis of change in HbA1C (%) in included trials using random effects model.

All 4 trials reported change in HbA1C from baseline to end of study period. We performed a random effects meta-analysis that included 915 participants assigned to GLP-1 analogue groups and 840 patients assigned to the sitagliptin groups (Table 2). The weighted mean reduction in HbA1C was larger for patients in GLP-1 analogues groups than for those in the sitagliptin groups (mean difference –0.41%, 95% CI –0.51 to –0.31) (Figure 3,Table 2). We found no evidence of significant heterogeneity in the analysis (I^2^ = 1%, P = 0.39). Subgroup analyses showed an HbA1C reduction in trials assessing exenatide (–0.49%, –0.73 to −0.25, I^2^ = 50%, P = 0.16) and liraglutide (–0.38%, –0.50 to –0.26, I^2^ = 0%, P = 0.64). The proportion of participants who achieved the HbA1C target (<7%) was higher in the GLP-1 analogues groups than in sitagliptin groups (relative risk 2.63, 95% CI 2.05 to 3.37, I^2^ = 0%, P = 0.45) (Table 2). The corresponding number needed to treat using the pooled odds ratio from the meta-analysis would be 5 (95% CI 4 to 6) [44].

**Table 2:** Summary of Meta-analyses of Outcomes in Patients with Type 2 Diabetes treated With GLP-1 analogues vs Sitagliptin.

Outcome	No. ofStudiesContributingData	Risk Ratio(95% CI),GLP-1analogues vsSitagliptin	Weighted MeanDifference(95% CI) inChange FromBaseline, GLP-1analogues vsSitagliptin	I^2^Heterogeneity,%	No. ofParticipantsWith DataAnalyzed forGLP-1analogues groups	No. of ParticipantsWith DataAnalyzed forSitagliptin groups
HbA1C	4		−0.41 (−0.51, −0.31)	0	915	840
Percentage ofpatientsachieved HbA1c<7%	3	2.63 (2.05, 3.37)		0	607	528
Fasting plasmaglucose level	4		−1.10 (–1.31, −0.89)	0	890	817
Weight loss	3		−1.55 (−1.98, −1.12)	0	590	521
Systolic bloodpressure	4		−0.83 (−3.00, 1.34)	71	588	517
Diastolic bloodpressure	4		0.07 (−1.29, 1.44)	57	629	559
Total cholesterol	3		−0.10 (−0.23, 0.02)	33	539	468
HDL	3		−0.01 (−0.03, 0.01)	0	539	468
LDL	1		−0.05 (−0.19, 0.09)	N/A	194	200
Triglyceride	1		0.21 (−0.05, 0.47)	N/A	191	198

N/A, not applicable; CI, confidence interval.

**Figure 4:**
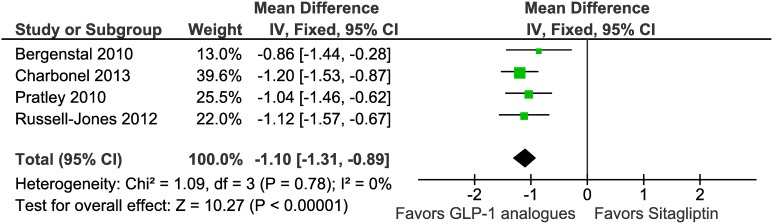
Meta-analysis of change in Fasting Plasma Glucose (mmol/L) in included trials using random effects model.

All 4 trials reported fasting plasma glucose (FPG). Random effects meta-analysis showed that there is a significant difference for reduction in FPG between patients in GLP-1 analogues groups and those in the sitagliptin groups (mean difference –1.10 mmol/L, 95% CI –1.31 to −0.89, I^2^ = 0%, P = 0.78) (Figure 4, Table 2). Subgroup analyses showed significant difference in FPG reduction between the exenatide group (−1.02 mmol/L, −1.38 to 0.67, I^2^ = 0%, P = 0.49) and sitagliptin group, and a significant reduction in liraglutide group compared to sitagliptin groups (–1.14 mmol/L, –1.40 to –0.88, I^2^ = 0%, P = 0.56) as well. None of the studies reported postprandial plasma glucose.

### Body weight

**Figure 5:**
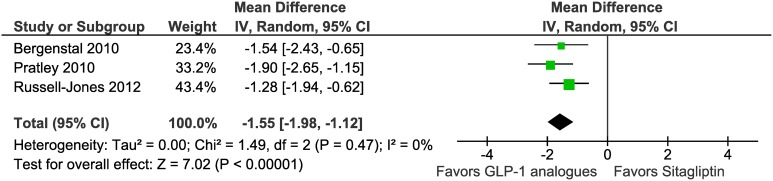
Meta-analysis of change in body weight (kg) of included trials using random effects model.

Three trials reported weight loss. We did a random effects meta-analysis including 590 participants assigned to GLP-1 analogues groups and 521 assigned to the sitagliptin groups. The intervention groups in the analysis received exenatide or liraglutide (Table 1). The weighted mean change in body weight was larger for patients in GLP-1 analogues groups than for those in the sitagliptin groups (mean difference –1.55 kg, 95% CI –1.98 to –1.12, I^2^ = 0%, P = 0.47) (Figure 5, Table 2). Subgroup analyses showed a weight reduction in trials assessing exenatide (–1.37 kg, –1.90 to –0.84, I^2^ = 0%, P = 0.65).

### Blood pressure and Lipid

Random effects meta-analysis didn’t show significant difference in reduction in blood pressure or lipid profile between GLP-1 analogues groups and Sitagliptin groups: mean difference for systolic blood pressure and diastolic blood pressure was –0.91 mmHg (–3.63 to 1.82) and −0.34 mmHg (−1.66 to 0.98) respectively (Table 2); and mean difference for total cholesterol, high-density lipoprotein (HDL), and low-density lipoprotein (LDL), and triglyceride were –0.10 mmol/L (–0.23 to 0.02), −0.01 mmol/L (−0.03 to 0.01), −0.05 mmol/L (−0.19 to 0.09) and 0.21 mmol/L (−0.05 to 0.47), respectively (Table 2). A repeat of meta-analysis using a high dose of a GLP-1 analogue (liraglutide 1.8 mg) showed a significant reduction in total cholesterol compared to sitagliptin: mean difference –0.16 mmol/L (–0.25 to −0.06, I^2^ = 0%, P = 0.63). Subgroup analyses showed that compared to sitagliptin, the exenatide group had a significant reduction in total cholesterol (–0.16 mmol/L, –0.29 to −0.03, I^2^ = 0%, P = 0.34) and no significant increase in HDL cholesterol (–0.02 mmol/L, –0.05 to 0.02, I^2^ = 0%, P = 0.68).

### Adverse events

Major hypoglycemia (Table S2) was reported in only 2 patients receiving GLP-1 analogues (liraglutide 1.2 mg QD); and there was no difference in reported minor to moderate hypoglycemia (Table S2) between GLP-1 analogues and sitagliptin (relative risk 1.35, 95% CI 0.71–2.58) (Table 3). Treatment with sitagliptin resulted in lower discontinuation rates (relative risk 2.89, 95% CI 1.42–5.87); nausea, vomiting, diarrhea, and constipation were also more common in patients receiving GLP-1 analogues than sitagliptin (Table 3). No difference in the incidence of urinary tract infection (UTI), upper respiratory tract infection (URTI), nasopharyngitis, and headache was evident between GLP-1 analogues and sitagliptin. Overall, sitagliptin were better tolerated, with lower absolute rates of adverse effects. Table 3 summarizes the findings of the main analyses for safety outcomes.

**Table 3:** Summary of meta-analyses of adverse events in patients with type 2 diabetes treated with GLP-1 analogues vs Sitagliptin.

Adverse event	No. of studiescontributing data	Relativerisk (95% CI)	I^2^Heterogeneity, %	Comparatorgroup (Event/Total)	
				GLP-1analogues	Sitagliptin
Withdrawal	3	2.89 (1.42 to 5.87)	0	31/629	10/548
Hypoglycemia	4	1.35 (0.71 to 2.58)	16	33/956	22/874
Nausea	3	3.14 (2.15 to 4.59)	1	112/629	32/548
Vomiting	3	2.60 (1.48 to 4.56)	0	47/629	16/548
Diarrhea	3	1.82 (1.24 to 2.69)	0	72/629	35/548
Constipation	3	2.50 (1.33 to 4.70)	0	40/629	13/548
Urinary tract infection	1	1.15 (0.48 to 2.76)	N/A	10/160	9/166
Upper respiratory tract infection	1	0.41 (0.17 to 1.04)	N/A	6/160	15/166
Nasopharyngitis	2	0.83 (0.57 to 1.22)	0	46/469	47/382
Headache	3	0.87 (0.61 to 1.23)	0	56/629	57/548

N/A, not applicable; CI, confidence interval.

## Discussion

### Explanation for findings

In this meta-analysis we assessed the efficacy and safety of incretin therapies using data from 4 trials comparing sitagliptin with GLP-1 analogue. The results demonstrate that compared to sitagliptin, the GLP-1 analogues, exenatide and liraglutide, are more efficacious in reducing HbA1C and body weight with similar efficacy in reducing blood pressure and changes in lipid parameters compared to sitagliptin. In terms of adverse effects, sitagliptin is better tolerated and has a lower incidence of gastrointestinal adverse events compared to GLP-1 analogues. Compared to GLP-1 analogues, sitagliptin treatment did not seem to increase the risk of hypoglycemia, although a previous meta-analysis showed that GLP-1 analogues did not similarly increase the risk of hypoglycemia [24]. In addition, sitagliptin did not appear to increase the risk of developing UTI, URTI, nasopharyngitis, and headache. Most trials lasted less than 26 weeks, limiting our assessment of long-term efficacy and safety. Serious or rare adverse events such as pancreatitis are not addressed in this meta-analysis because they are hard to detect from RCTs with a relatively small sample size. The included trials did not provide enough data to compare GLP-1 analogues and sitagliptin regarding major cardiovascular events. Only ongoing prospective head to head clinical trials specifically designed to study the effects of cardiovascular events will provide further information in this respect.

Based upon our study, GLP-1 analogues demonstrate superiority in clinical efficacy (−0.41%) and weight loss (−1.55 kg) but have a higher incidence of gastrointestinal events and require delivery by subcutaneous injection. On the other hand, sitagliptin is less efficacious but has fewer gastrointestinal side effects and is available by oral administration [18]. Incretin therapies will play an increasing role in management of patients with T2DM as add-on agents to metformin therapy or as recommended options for three drug combinations that includes basal insulin. Sitagliptin might be considered as a more favorable option for early intervention in T2DM management as the initial add on therapy to metformin in patients whose glycemic control is closer to target goals and GLP-1 analogues might be preferred in over-weight or obese patients who require better glycemic control.

### Assessment of quality of included studies

Inadequate randomization and attrition bias could result in overestimating effects of an intervention. The study by Charbonel et al., did not blind the participants or account for the intention to treat population in their results and analyses [32]. These aspects weakened the internal validity of our findings. Since only trials that used clinically relevant doses given for clinically relevant treatment periods were included in our meta-analysis, the results can be extrapolated to clinical practice.

### Strengths and Limitations

The strengths of this meta-analysis are related to the incorporation of direct evidence from recently published head to head trials, the variety of outcomes assessed, and the investigation of plausible clauses of heterogeneity by sensitivity analyses. However, some limitations should also be recognized.

First, we only included 4 head to head trials based on our rigorous inclusion criteria. Three head to head studies were excluded due to their short study period of 2, 4, and 8 weeks, respectively [28], [29], [33]. Furthermore, intervention effects of different doses and formulations were not examined in subgroup analysis due to the paucity of available data, and our conclusions regarding exenatide or liraglutide interventions compared to sitagliptin in subgroup analysis were not robust enough because of the small number of relevant trials. Secondly, there was considerable variation in the risk of bias across the included studies, although exclusion of trials at high risk of bias in a sensitivity analysis did not alter the results of the main analysis. Third, long term efficacy and safety of GLP-1 analogues and sitagliptin was not compared; and rare, serious adverse events such as pancreatitis or renal failure [22] were not evaluated. Lastly, head-to-head studies of DPP-4 inhibitors in the literature included primarily sitagliptin as the comparator. Other DPP4 inhibitors were not included in this meta-analysis.

## Conclusion

This meta-analysis included 4 head-to-head studies comparing the short-term efficacy and safety of GLP-1 analogues and sitagliptin. The results demonstrate that compared to sitagliptin, GLP-1 analogues are more efficacious for glycemic control and weight loss, but not better in reducing blood pressure and lipid profile; and GLP-1 analogues have a higher incidence of gastrointestinal adverse events and similar hypoglycemic events compared to sitagliptin. For less common adverse events, GLP-1 analogues and sitagliptin have a similar incidence of headache, UTI, URTI, and nasopharyngitis. If weight loss is not a particular concern and only a small decrease in A1C is required, a DPP-4 inhibitor may be better choice. Future long-term head-to-head RCTs assessing the GLP-1 analogues versus sitagliptin should be designed to provide a definitive answer regarding the place of the two classes of agents in the treatment algorithm.

## Supporting Information

Checklist S1 **PRISMA checklist.**
(DOC)

Table S1 **Summary of efficacy from included studies.**
(DOCX)

Table S2 **Summary of adverse events from included studies.**
(DOCX)
