# Supplementary material for: Comparison of GLP-1 Analogues versus Sitagliptin in the Management of Type 2 Diabetes: Systematic Review and Meta-Analysis of Head-to-Head Studies
Source: PLoS One. 2014 Aug 4;9(8):e103798. doi: 10.1371/journal.pone.0103798 (PMC4121242; doi:10.1371/journal.pone.0103798)
Supplement: Table S1 — Summary of efficacy from included studies. (DOCX) [file pone.0103798.s002.docx]

**Table S1.** Summary of efficacy from included studies

| Study (First aughor, year) | Weight Loss (kg) | HbA1C reduction (%) | The proportion of participants who achieved HbA1C <7% | Fasting plasma glucose (FPG) reduction (mmol/) | 2h postprandial glucose (PPG) reduction (mmol/l) | Change in Heart/pulse Rate (beats/min) | Change in systolic blood pressure (mm/Hg) | Change in diastolic blood pressure (mm/Hg) | Change in total cholesterol (mmol/L) | Change in HDL (mmol/L) | Change in LDL (mmol/L) | Change in triglyceride (mmol/L) |
| --- | --- | --- | --- | --- | --- | --- | --- | --- | --- | --- | --- | --- |
| **Bergenstal 2010*** CT00637273  **(Duration-2)** | I/C 160/165 | I/C 159/162 | I/C 160/166 | I/C 155/161 | N/A | N/A | I/C 160/163 | I/C 160/163 | I/C 146/147 | I/C 146/147 | N/A | N/A |
|  | I **-2.31 (0.323) <SD4.09>** | I **-1.55 (0.100) <SD1.26>** | I **58.8%** | I **-1.76 (0.21) <SD2.61>** | N/A | N/A | I **-3.6 (0.97) <SD12.27>** | I **-1.4 (0.57) <SD 7.21>** | I **-0.02 (0.07) <SD 0.85>** | I **0.05 (0.02) <SD 0.24>** | N/A | N/A |
|  | C **-0.77 (0.322) <SD4.12>** | C **-0.92 (0.099) <SD1.26>** | C **30.7%** | C **-0.9 (0.21) <SD2.66>** | N/A | N/A | C  **0.2 (0.95) <SD12.13>** | **C -0.4 (0.57) <SD 7.28>** | **C 0.08 (0.06) <SD 0.73>** | **C 0.05 (0.02) <SD 0.24>** | N/A | N/A |
| **Charbonel 2013^a^** NCT01296412 | N/A | I -1.2 {SD1.02} | N/A | I -2.3 {SD2.17} | N/A | N/A | N/A | N/A | N/A | N/A | N/A | N/A |
|  | N/A | C -0.8{SD1.01} LSM, 95%CI | N/A | C -1.1{SD2.17}  LSM, 95%CI | N/A | N/A | N/A | N/A | N/A | N/A | N/A | N/A |
| **Pratley 2010***NCT00700817 (1860-LIRA-DPP-4 Study Group) | I_1_/ I_2_/C 215/214/215 | I_1_/ I_2_/C 211/214/210 | I_1_/ I_2_/C 221/218/219 | I_1_/ I_2_/C 210/212/210 | N/A | I_1_/ I_2_/C 212/214/211 | I_1_/ I_2_/C 213/214/213 | I_1_/ I_2_/C 213/214/213 | I_1_/ I_2_/C 194/202/201 | I_1_/ I_2_/C 194/201/201 | I_1_/ I_2_/C 194/202/200 | I_1_/ I_2_/C 191/199/198 |
|  | I_1_ **-2.86 (0.27) <SD 3.96>** | I_1_ **-1.24 (0.07) <SD 1.02>** | I_1_ **43%** | I_1_ **-1.87 (0.15) <SD 2.17>** | N/A | I_1_ **2.32 (0.59) <SD 8.59>** | I_1_ **-0.55 (0.89) <SD 13.23>** | I_1_ **-0.71 (0.60) <SD 8.92>** | I_1_ **-0.03 (0.06) <SD 0.84>** | I_1_ **0.00 (0.01) <SD 0.14>** | I_1_ **0.08 (0.05) <SD 0.70>** | I_1_ **-0.19 (0.1) <SD 1.38>** |
|  | I_2_ **-3.38 (0.27) <SD 3.95>** | I_2_ **-1.5 (0.06) <SD 0.88>** | I_2_ **55%** | I_2_ **-2.14 (0.15) <SD 2.18>** | N/A | I_2_ **3.94 (0.58) <SD 8.48>** | I_2_ **-0.72 (0.89) <SD 13.14>** | I_2_ **0.07 (0.59) <SD 8.71>** | I_2_ **-0.17 (0.05) <SD 0.71>** | I_2_ **0.00 (0.01) <SD 0.14>** | I_2_ **0.05 (0.05) <SD 0.71>** | I_2_ **-0.43 (0.09) <SD 1.27>** |
|  | **C -0.96 (0.27) <SD3.96>** | **C -0.9 (0.07) <SD1.01>** | **C 22%** | **C -0.83 (0.15) <SD 2.17>** | N/A | **C -0.64 (0.59) <SD 8.57>** | **C -0.94 (0.89) <SD 13.17>** | **C -1.78 (0.60) <SD 8.88>** | **C -0.02 (0.05) <SD 0.71>** | **C 0.00 (0.01) <SD 0.14>** | **C 0.13 (0.05) <SD0.71>** | **C -0.40 (0.09) <SD1.27>** |
| **Russell-Jones 2012*** NCT00676338 **(Duration-4)** | I/C 215/141 | I/C 218/142 | I/C 226/143 | I/C 198/120 | N/A | N/A | I/C 215/141 | I/C 215/141 | I/C 199/120 | I/C 199/120 | N/A | N/A |
|  | I **-2.04 (0.21) <SD 3.08>** | I **-1.53 (0.07) <SD 1.03>** | I **64.2%** | I **-2.25 (0.14) <SD 1.97>** | N/A | I 1.5 (10.0) <SD 157.48> | I **-1.25 (0.79) <SD 11.58>** | I **-0.5 (0.51) <SD 7.48>** | I **-0.24 (0.06) <SD 0.85 >** | I **0.01 (0.01) <SD 0.14 >** | N/A | N/A |
|  | **C -0.76 (0.26) <SD3.09>** | **C -1.15 (0.08) <SD0.95>** | **C 45.5%** | **C -1.13 (0.18) <SD1.97>** | N/A | C 0.5 (SE9.7) <SD 128.84> | **C -1.81 (0.96) <SD11.40>** | **C -0.45 (0.62) <SD7.36>** | C **-0.01 (0.08) <SD 0.88>** | C **0.04 (0.02) <SD 0.22>** | N/A | N/A |

I/C, No. of Patients analyzed in intervention and control group obtained from [www.clinicaltrials.gov](http://www.clinicaltrials.gov) ; *Data obtained from Clinicaltrials.gov

LSM，least square mean; (SE); [SD] calculated from mean, sample size, and lower/upper limit of 95% confidence interval; <SD> calculated from standard error; {SD} imputed from the other trial assessing liraglutide [Pratley 2011] with a reasonably high standard deviation.

**^a^**only data from the first 12 weeks were used to because glimepiride was added to sitagliptin groups for patients with inadequately controlled glucose after 12 weeks.
