# Supplementary material for: Comparison of GLP-1 Analogues versus Sitagliptin in the Management of Type 2 Diabetes: Systematic Review and Meta-Analysis of Head-to-Head Studies
Source: PLoS One. 2014 Aug 4;9(8):e103798. doi: 10.1371/journal.pone.0103798 (PMC4121242; doi:10.1371/journal.pone.0103798)
Supplement: Table S2 — Summary of adverse events from included studies. (DOCX) [file pone.0103798.s003.docx]

**Table S2.** Summary of adverse events from included studies

| Study (First author, year) | Withdrawal due to side effect n (%) | Hypoglycaemia n (%) Minor^a^ | Hypoglycaemia n (%) Major | Nausea n (%) | Vomiting n (%) | Diarrhea n (%) | Constipation n (%) | UTI n (%) | URTI n (%) | Nasopharyngitis n (%) | Headache n (%) |
| --- | --- | --- | --- | --- | --- | --- | --- | --- | --- | --- | --- |
| **Bergenstal 2010 NCT00637273 (Duration-2)** | I 11 | I 2 | I 0 | I 38/160 (24) | I 18/160 (11) | I 29/160  (18) | I 9/160 (6%) | I 10 (6%) | I 6 (4%) | N/A | I 15(9) |
|  | C 5 | C 5 | C 0 | C 16/166 (10) | C 4/166 (2) | C 16/166 (10) | C 3/166 (2%) | C 9 (5%) | C 15 (9%) | N/A | C 15(9) |
| **Charbonel 2013*** NCT01296412 | N/A | I 8 | I 0 | N/A | N/A | N/A | N/A | N/A | N/A | N/A | N/A |
|  | N/A | C 6 | C 0 | N/A | N/A | N/A | N/A | N/A | N/A | N/A | N/A |
| **Pratley 2010** NCT00700817 (1860-LIRA-DPP-4 Study Group) | I_1_14 | I_1_ 17/221 | I_1_ 1/221 | I_1_ 46 (21) | I_1_ 17 (8) | I_1_ 16 (7) | I_1_ 10 (5) | N/A | N/A | I_1_ 21 (10) | I_1_ 20 (9) |
|  | I_2_ 15 | I_2_ 37/218 | I_2_ 0 | I_2_ 59 (27) | I_2_ 21 (10) | I_2_ 25 (11) | I_2_ 11 (5) | N/A | N/A | I_2_ 28 (10) | I_2_ 25 (11) |
|  | C 4 | C 11/219 | C 0 | C 10 (5) | C 9 (4) | C 10 (5) | C 6 (3) | N/A | N/A | C 26 (12) | C 22 (10) |
| **Russell-Jones 2012** NCT00676338 **(Duration-4)** | I 6 | I 5/248 (2%) | I 0 | I 28/248 (11.3) | I 12/248 (4.8) | I 27/248 (10.9) | I 21/248 (8.5) | N/A | N/A | I 19 (7.7) | I 20 (8.1) |
|  | C 1 | C 0 | C 0 | C 6/163 (3.7) | C 3/163 (1.8) | C 9/163 (5.5) | C 4/163 (2.5) | N/A | N/A | C 16(9.8) | C 15 (9.2) |

^a^ No major hypoglycemia events.

* only data of the first 12 weeks were recorded because glimepiride was added to sitagliptin groups for patients with inadequately controlled glucose after 12 weeks.
